# Identification of Non-Tuberculous Mycobacteria in Iberian Lynx (*Lynx pardinus*) and Their Impact on Its Health

**DOI:** 10.3390/vetsci12060527

**Published:** 2025-05-29

**Authors:** Natalia Jiménez-Pizarro, Beatriz Serrano, Jorge Peña, Rafael Barrera, María Gil-Molino, David Risco, Javier Hermoso-de-Mendoza

**Affiliations:** 1 Animal Health Department, Faculty of Veterinary, University of Extremadura, 10003 Cáceres, Spain; nataliajp@unex.es (N.J.-P.); magilmo84@gmail.com (M.G.-M.); jhermoso@unex.es (J.H.-d.-M.); 2 Life Lynxconnect Project, FOTEX S.L., 06011 Badajoz, Spain; beatriz.serrano@fotex.es; 3 Natural Environment Area, Public Management Society of Extremadura (GPEX), Junta de Extremadura, 06187 Mérida, Spain; jorgepmartinez@gmail.com; 4 MINVET Group, Animal Medicine Department, Faculty of Veterinary, University of Extremadura, 10003 Cáceres, Spain; rabacha@unex.es; 5 Animal Medicine Department, Faculty of Veterinary, University of Extremadura, 10003 Cáceres, Spain

**Keywords:** lynx, *Lynx pardinus*, non-tuberculous mycobacteria, *Mycobacterium lentiflavum*, *Mycobacterium gordonae*

## Abstract

The Iberian lynx (*Lynx pardinus*) population has been increasing in recent years thanks to the collaboration of several associations within the framework of the Life Lynxconnect project, which promotes captive breeding for the subsequent release of specimens into the environment. It is therefore important to know their population status, the diseases to which these animals are exposed, and how they affect their repopulation. In this sense, this study aims to study how the presence of mycobacteria affects the lynx population in Extremadura (southwest of Spain). To this end, blood samples, tracheal swabs, and tracheobronchial washings from live-captured lynxes, as well as mediastinal and mesenteric lymph nodes from roadkill animals, were collected. The association between the presence of mycobacteria and various factors—including body condition, age, sex, and blood parameters—was subsequently evaluated. Up to date, our findings show the absence of tuberculosis (TB) in all lynxes tested, while they seem to be reservoirs of non-tuberculous mycobacteria (NTM), mainly *M. lentiflavum*, the most isolated species. Nonetheless, these mycobacteria appear to have no significant effect on the health condition of the animals.

## Simple Summary

The Iberian lynx (*Lynx pardinus*) population is growing thanks to conservation initiatives such as the Life Lynxconnect project. To support this recovery, this study analyzed samples from live-captured and deceased lynxes in Extremadura (southwestern Spain) to assess the presence of mycobacteria and their impact on health. Tuberculosis was not detected, but non-tuberculous mycobacteria, primarily *M. lentiflavum*, were detected, which do not appear to harm lynx health.

## 1. Introduction

The genus *Mycobacterium* contains 272 species and 24 validly published subspecies [1]. Mycobacteria from the *Mycobacterium tuberculosis* complex (MTC), such as *M. bovis*, *M. caprae*, or *M. tuberculosis*, are the best known due to their effects on human and animal health [2]. On the other hand, within the genus *Mycobacterium*, all mycobacteria other than the MTC, *M. leprae*, and *M. ulcerans* are called non-tuberculous mycobacteria (NTM) or atypical mycobacteria.

The NTM group includes more than 150 different species, with the *Mycobacterium avium* complex (MAC) being the most well-known due to its ability to cause opportunistic infections, particularly in immunocompromised individuals [3]. NTM are generally believed to be acquired from the environment through ingestion, inhalation, and skin contact, resulting in lymphadenitis, pulmonary and disseminated infections, and skin and soft tissue infections [4]. Most of them are widely distributed in the environment, being present in soil, water, dust, in living organisms, or even food [5], although the natural reservoirs of some species such as *M. kansasii* and *M. xenopi* remain unknown [6].

These kinds of infections caused by the NTM are emerging in the western world due to several factors like an increase in mycobacterial infection sources in the environment or the improvements in laboratory detection techniques [4]. Some subspecies of the MAC, such as *Mycobacterium avium* subsp. *paratuberculosis*, have been associated with human diseases like Crohn’s disease [3], type 1 diabetes mellitus, or multiple sclerosis [7].

However, in veterinary medicine, except for *M. avium* subsp. *paratuberculosis*, the presence of NTM is generally not specifically investigated [8]. Thus, isolation of NTM is usually a secondary finding of MTC research, since active surveillance for the detection of NTM is not contemplated in livestock health control campaigns and wildlife surveillance programs in general [2].

In fact, there are few studies identifying NTM in wildlife in Extremadura, a region located in south-western Spain. The first reported identifications were *M. fortuitum* in black kite (*Milvus migrans*) and *M. avium* in wild boar (*Sus scrofa*) [9]. In Spain, other NTM have been identified as *M. avium* subsp. *avium* and *M. avium* subsp. *hominissuis*, which have been detected in badger (*Meles meles*) and wild boar [10,11]; *M. chelonae*, *M. intracellulare*, and *M. avium*, in wild boar from Extremadura [12]; and *M. intracellulare* and *M. interjectum* which were found in red deer (*Cervus elaphus*) [13]. Members of the MAC have been reported as well in roe deer (*Capreolus capreolus*) and foxes (*Vulpes vulpes*) [11].

However, NTM have not been reported in other wildlife species like the Iberian lynx (*Lynx pardinus*). Regarding tuberculosis (TB), a first case in free-living Iberian lynx from Doñana National Park in Spain was published by Briones et al., in 2001 [10,11] and there is some more description about MTC isolates in lynx. In fact, according to the Visavet database *M. bovis* has been isolated from seven lynxes from 1999 to 2022 spolygotyped as SB1232 (n = 3), SB0295 (n = 2), and SB1258 (n = 1) from Huelva, in Andalusia Region, and SB1263 (n = 1) from Toledo, in Castilla–La Mancha Region (www.visavet.es/mycodb, accessed on 20 May 2025). However, no studies have been conducted to determine its health impact.

The population of Iberian lynx, a species that 20 years ago was categorized as critically endangered by the International Union for Conservation of Nature and considered as lost by many experts, is growing today. In fact, while the Iberian lynx population in Extremadura was estimated at 116 individuals in 2021 [14], it increased to 137 in 2022 [15], the year in which the samples analyzed in this study were collected (www.miteco.gob.es, accessed on 20 May 2025). One of the main concerns about Iberian lynx repopulation is to avoid sanitary problems caused by infectious and parasitary pathogens that may lead to mortality in this species. Viral infections such as parvovirus, canine distemper virus (CDV), feline leukemia virus (FeLV), and Aujeszky’s disease (SuHV-1) that could be fatal in the Iberian lynx were analyzed in 2021 and the results indicated that infectious diseases did not pose a threat to the constant growth of the Iberian lynx population in this region [16]. In fact, in the Doñana National Park (southern Spain) there was a documented serious FeLV epidemic in 2006 that caused a high mortality rate in Iberian lynxes [17]. In this sense, the MTC and NTM are also pathogens that could play an important role, since Iberian lynx is known to predate mainly on rabbits and occasionally on wild ungulates, both involved in the propagation cycle of both the MTC and NTM [18].

The objective of the current study was to analyze and identify the presence of mycobacteria in the Iberian lynx population to know if, at present, the risks of TB and other mycobacteriosis can be ruled out in this species. In addition, it was intended to assess the effects of the presence of mycobacteria on the health of lynxes, to know its impact on the reintroduction of this animal species in nature.

## 2. Materials and Methods

Of the total of 64 lynxes born in captivity analyzed in this study, 59 were captured alive and five of them were road-killed. The samples came from individuals captured in the livestock areas of southern Badajoz, while only nine lynxes were captured in the province of Cáceres (northern Extremadura) (Table 1). These samples were analyzed by veterinarians from FOTEX S.L., GPEX, and the UEX Mycobacteria and Brucellae Laboratory (Animal Health Department, School of Veterinary Sciences, University of Extremadura, Cáceres, Extremadura, Spain).

Free-ranging lynxes were captured using commercial cage traps (Tomahawk models 108 and 207, Tomahawk Live Trap Co., Tomahawk, Hazelhurst, WI, USA) baited with rabbits (*Oryctolagus cuniculus*). Once captured, they were anesthetized using a mixture of dexmedetomidine–midazolam–ketamine. Anesthetized Iberian lynxes underwent a complete routine health evaluation. Blood (1–3 mL) was obtained from the saphenous vein and collected in EDTA-coated tubes and/or serum separator tubes (Aquisel, Selecta Group, Barcelona, Spain). Lynxes were identified by microchip reading and spot pattern. The lynxes found dead were subjected to a field postmortem examination, but no hematological or blood biochemical analyses could be performed. In addition, data could not be taken from two of them due to the state of decomposition of their corpses.

The samples of choice to analyze mycobacteria were tracheal swabs and tracheobronchial washings from the live-captured lynxes as well as mediastinal and mesenteric lymph nodes from the two road-killed lynxes.

Whenever possible, epidemiological information about each individual animal was recorded, including age (yearlings: <1 year old; subadults: 1 to 3 years old; adults: 3 to 10 years old), sex, physical exploitation/body condition, georeferenced location, and other parameters like lymph node palpation to determinate lymphadenomegaly.

### 2.1. Real-Time PCR

Real-time PCR was used as an initial screening method to determine the presence of the MTC in swabs, tracheobronchial washes, and other biological samples received.

The cotton head of each nasopharyngeal swab was rehydrated in 1.5 mL of distilled water, vortexed for a few seconds, and subsequently left to rest for 15 min. An amount of 1 mL of suspension was used for culture and the other 0.5 mL was used for qPCR.

Regarding tracheobronchial washings, 4 mL were used, 2 for qPCR and 2 for culture.

Suspensions were centrifuged for 20 min at 13,000 rpm. In the case of tracheobronchial lavages, the supernatant was discarded, and a swab was inserted in the sediment which was then used to extract the DNA with the kit.

Lymph nodes were homogenized with distilled water in a stomacher for 4 min. Then, for the extraction of DNA from all the samples, we followed the manufacturer’s protocol of the Patho gene-spinTM Kit (iNtRON Biotechnology, Seongnam, Republic of Korea).

To determine the presence of mycobacteria of MTC DNA, a specific real-time PCR assay targeting the IS1561′ locus was carried out using a pair of primers IS1561-F (5′ GATCCAGGCCGAGAGAATCTG 3′) and IS1561-R (5′ GGACAAAAGCTTCGCCAAAA 3′) and a IS1561-Probe (5′ ACGGCGTTGATCCGATTCCGC 3′) (Eurogentec, Seraing, Belgium) [19] with an Applied Biosystems QuantStudio 3 thermal-cycler (Thermo Fisher Scientific, Waltham, MA, USA). IS1561′ locus sequence is used as is present in all the MTC members except in *M. microti*. The PCR conditions were as follows: an initial activation for 2 min at 95 °C, followed by 45 amplification cycles consisting of 5 s of denaturation at 95 °C and 2 min of hybridization at 60 °C [20]. A strain of *M. bovis* (SB0121) was used as a positive control and non-template samples were used as negative controls.

### 2.2. Mycobacteria Culture

For the mycobacteria culture from nasopharyngeal swabs, the steps were as follows: 1 mL was subjected to a treatment with bovine serum albumin for 3 min to enhance the mycobacteria growth, and a decontamination treatment with N-acetyl-L-cysteine–sodium hydroxide solution (BBL MycoPrep, BD) for 15 min and with di-sodium hydrogen phosphate anhydrous (Panreac) as a stop solution. Then, 0.5 mL were cultured in liquid media (BD BBL Mycobacteria Growth Indicator Tube [MGIT], Becton and Dickinson, Franklin Lakes, NJ, USA), because MGIT has a higher sensitivity for mycobacteria detection and offers faster results compared with solid media cultures. Culture media were incubated using a BACTEC MGIT 960 semi-automatic culture incubator (Becton and Dickinson, Franklin Lakes, NJ, USA) at 37 °C under aerobic conditions for 42 days.

After centrifugation of the tracheobronchial lavage samples, we obtained 1 mL of the sediment, which was cultured following the same steps described in the previous paragraph.

Regarding lymph nodes, 7.5 mL of the homogenate was mixed with 7.5 mL of the same decontaminant and 35 mL of stop solution. The samples were centrifuged at 3000 rpm for 20 min. The supernatant was decanted and 0.5 mL of the pellet was inoculated in the same liquid culture.

### 2.3. Mycobacterial Isolation and DNA Extraction

DNA extraction for mycobacterial identification was performed by taking bacterial growth from the liquid medium. For this purpose, 1.5 mL of the culture medium was collected in an Eppendorf vial and centrifuged for 30 s. The supernatant was discarded, and the pellet was resuspended in 250 µL of ultrapure water. The suspensions were inactivated at 100 °C for 10 min and centrifuged to clean the DNA in the supernatant.

### 2.4. Mycobacterium spp. Identification by Convencional PCR

Conventional PCR to identify genus Mycobacterium and the MTC was carried out following standard methods [21] using the primers TB1-F (5′ GAACAATCCGGAGTTGACAA 3′), TB1-R (5′ AGCACGCTGTCAATCATGTA 3′), MYCGEN-F (5′ AGAGTTTGATCCTGGCTGGCTCAG 3′), and MYCGEN-R (5′ TGCACACACAGGCCACAAGGGA 3′) (Eurogentec, Seraing, Belgium) [22], the polymerase DreamTaq Green PCR Master Mix (Thermo Scientific, Waltham, MA, USA) and the DNAs previously obtained. The PCR conditions consisted of an initial denaturation at 95 °C for 5 min; 35 amplification cycles including 30 s of denaturation at 95 °C, 1 min of annealing at 60 °C and 2 min of primer extension at 72 °C; and finally, an elongation step of 10 min at 72 °C.

The products were analyzed by electrophoresis in 1.5% agarose gel (Condalab, Spain). The presence of a unique 1030 base pairs (bp) electrophoretic band indicated species of NTM and two DNA fragments at 1030 bp and 370 bp corresponded to the MTC.

### 2.5. NTM Identification by PCR and Restriction Enzyme Analysis and Interpretation (PRA-hsp65)

To identify the different species of NTM isolated, a fragment (441-bp) of the 65-kDa heat shock protein gene (hsp65) was amplified by two specific primers Tb11 (5′ ACCAACGATGGTGTGTCCAT 3′) and Tb12 (5′ CTTGTCGAACCGCATACCCT 3′) (Eurogentec, Seraing, Belgium) [23]. The PCR conditions were as follow: initial denaturation at 94 °C for 5 min; 45 amplification cycles including 1 min of denaturation at 94 °C, 1 min of annealing at 60 °C, and 1 min of primer extension at 72 °C; and an elongation step of 10 min at 72 °C [12,23].

The hsp65 fragment was digested with BstEII and HaeIII (Takara Biotechnology Inc., Dalian, China) restriction enzymes [23]. The fragments obtained were detected in a 3% agarose gel (Condalab, Spain) and compared with a 20–500 bp molecular weight marker (Thermo Fisher Scientific, Waltham, MA, USA). Results were interpreted with the help of an algorithm described by Chimara et al., in 2008 [24]. To avoid confusion with primer and primer-dimer bands, restriction fragments shorter than 50 bp were disregarded.

### 2.6. Blood Analysis

The Life Lynxconnect project carried out blood tests. A 5-population automatic blood analyzer (ProCyte DX, IDEXX^®^, Tokyo, Japan) was used for hematology, in which the following parameters were analyzed: total red blood cell count (RBC), hematocrit value, hemoglobin concentration, mean corpuscular volume (MCV), mean corpuscular hemoglobin concentration (MCHC), total and differential leukocyte count (WBC), and platelet count. In addition, blood smears were taken for morphological study of blood cells, observed by light microscopy (×40 and ×100; Diff-Quik staining method, QCA Laboratory).

Plasma biochemistry was performed in an automatic blood biochemistry analyzer (Spin 200E, Spinreact^®^, Barcelona, Spain), using specific commercial kits from Spinreact^®^ Laboratories. Plasma concentrations of glucose, total protein, albumin, urea, creatinine, calcium, phosphorus, total bilirubin, direct bilirubin, indirect bilirubin, alkaline phosphatase, alanine aminotransferase (ALT/GPT), aspartate aminotransferase (AST/GOT), cholesterol, and triglycerides were determined in all patients. The albumin/globulin ratio was subsequently calculated. Plasma concentrations of sodium, potassium, and chloride were determined by an automatic electrolyte analyzer (EasyElectrolytes, Medica^®^, Bedford, MA, USA).

Plasma electrophoresis was carried out in cellulose acetate, using a commercial kit for plasma proteins (Seleo Engineering^®^, Naples, Italy) in automated equipment (Selvet 24^®^). Buffer solution (pH 8.8), staining solution (Ponceau red), and decolorizing solution (citric acid, sodium phosphate, and sodium azide), provided by the kit manufacturer, were used. Protein migration was performed at 10 mA for 12 min. The electrophoresis result was expressed in g/dL.

### 2.7. Statistical Analysis

Statistical analyses were performed using IBM SPSS Statistics 22 software. Associations between the presence of mycobacteria and qualitative variables (age, sex, and presence of lymphadenomegaly) were analyzed using Pearson’s chi-square test while the associations between the presence of mycobacteria and body condition (quantitative variable) were analyzed using the non-parametric Kruskal–Wallis test. Firstly, the Shapiro–Wilk normality test (n < 50) was performed to assess if the blood parameters followed a normal distribution. Then, to know if there were significant differences in the blood parameters according to the presence of mycobacteria, the Student’s *t*-test was performed for those parameters with normal distribution and the Mann–Whitney U-test for those with non-normal distribution. Those comparisons with *p* < 0.05 were considered statistically significant.

## 3. Results

All the samples analyzed resulted negative for MTC identification by real-time PCR. Regarding the cultures, 48.44% of cultures (n = 31) had growth and were analyzed following conventional PCRs to identify genus Mycobacterium and the MTC. After that, it was observed that no sample had positive results for the MTC but a total of 87.09% (n = 27) of isolates were identified as belonging to the genus (NTM).

**Figure 1:**
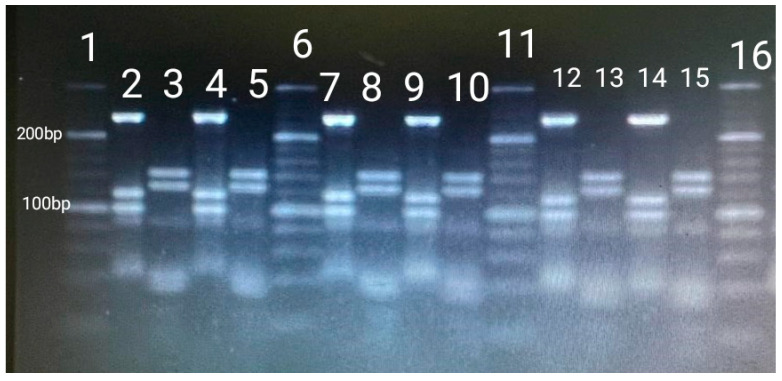
BstEII and HaeIII fragment lengths (base pairs) from *M. lentiflavum*. Columns 1, 6, 11, and 16: 20 bp ladder. Columns 2, 4, 7, 9, 12, and 14: Sizes of fragments of BstEII (235 bp/120 bp/100 bp). Columns 3, 5, 8, 10, 13, and 15: Sizes of fragments of HaeIII (145 bp/130 bp).

After comparing our restriction fragments patterns with the PRA- hsp65 patterning algorithm described [24] it was noted that the most frequently identified mycobacterium was *M. lentiflavum* with a 77.78% (n = 21) of the total (Figure 1), followed by *M. gordonae* identified in only four cultures. Lastly, in two of the cultures no mycobacteria could be identified, which could be due to contamination.

No association was detected between the detection of NTM and age group/class (*p* = 0.893), sex (*p* = 0.264), body condition (*p* = 0.843), or existence of lymphadenomegaly (*p* = 0.075) even though enlarged lymph nodes were observed in five lynxes, *M. lentiflavum* being detected in three of them and *M. gordonae* in one of them.

On the other hand, the effect of the presence of mycobacteria on the hematology and blood biochemistry of all lynxes captured alive was studied (Table 2), and no significant differences were found.

## 4. Discussion

In this study, no MTC mycobacteria were identified in the lynxes analyzed, despite Extremadura being an area of Spain where TB has been described in cattle and wildlife, which may suggest that the lynx has little interaction with these animals, perhaps due to its solitary nature [25]. Furthermore, MTC mycobacteria infections in lynxes may be rare, as, from 2002 to date, it has been identified only in one lynx in the province of Toledo (central Spain) (https://www.visavet.es/mycodb, accessed on 30 November 2023). Nevertheless, this may be also due to the fact that there are few studies of TB in Iberian lynx populations. However, the information gained in this study is novel as the presence of NTM in the Iberian lynx population, although expectable as ubiquitous bacteria, has not been described so far. Our findings indicate that *M. lentiflavum*, characterized by slow growth of tiny yellow colonies [26], is a common mycobacterium in the lynx population from the studied area. As with other NTM, *M. lentiflavum* has been isolated from soil and water samples around the world [27] and even from raw buffalo (*Bubalus bubalis*) milk [28]. On the other hand, *M. gordonae* has been largely isolated from water resources, because it has few nutrition requirements and a high resistance and tolerance against chlorine [29]. These findings suggest that water can be a source of infection of both mycobacteria for animals and people. In fact, in the Serengeti ecosystem, Tanzania, where humans and animals live closely together and many areas lack safe drinking water, *M. lentiflavum* has been isolated from six samples from cattle, three from buffalo specimens, one from Thompson’s gazelle (*Eudorcas thomsonii*) and one from a warthog (*Phacochoerus africanus*) [30].

*M. lentiflavum* was considered in 2016 among the new species of recently described NTM, and few cases had been published to that date after the first description of the infection in 1997 [31]. Nevertheless, unlike *M. gordonae*, a species of low pathogenicity which rarely causes disease in humans [32], *M. lentiflavum* is an NTM that causes infectious diseases of humans, such as cervical lymphadenitis, lung disease, and disseminated infections [33,34]. In fact, this mycobacterium has also been isolated from tracheobronchial lavage specimens in humans [35]. These results point to tracheobronchial lavages as samples of choice for the detection of mycobacteria. Lymphadenomegaly has been repeatedly observed in humans infected by this pathogen [36] especially in young children [34,37]. It agrees with our results, since in four of the five lynxes that presented lymphadenomegaly it was associated with NTM infections.

In addition to affecting humans, this mycobacterium has been recently isolated from red deer, roe deer, and wild boar, most of which had tuberculous lesions in retropharyngeal nodes [2]. However, unlike the Iberian lynx, several different NTM have been isolated from these species, being *M. avium* subsp. *avium* and *M. avium* subsp. *hominissuis* from the MAC, *M. nonchromogenicum* from the *M. terrae* complex, *M. lentiflavum* from the *M. simiae* complex, and *M. chelonae* the species most frequently isolated [2,12]. The difference between finding almost exclusively *M. lentiflavum* in lynxes in our study and the diversity of NTM found in other wild hosts may be related to their behavior. Wild boar, red deer, or roe deer habits imply a very close interaction between them as they share mud, water, and food areas with each other and with cattle, thus favoring the transmission of contagious agents such as NTM between them. Behavioral patterns of wild boar such as rooting and wallowing during rut also exemplify this interaction [38,39]. However, lynxes lack wallowing or rooting habits, being generally solitary animals, distributed throughout the territory according to sex (with males occupying larger territories than females), the abundance of rabbits, and the quality of the habitat. An amount of 94% of the Iberian lynx’s diet is based on wild rabbits (*Oryctolagus cuniculus*) [25], although they can also feed on other prey such as red-legged partridges (*Alectoris rufa*), hares (*Lepus granatensis*), different species of birds, rodents, and, on rare occasions, carrion. Furthermore, while *M. gordonae* has been described in small mammals [40,41], *M. lentiflavum* has not been described in rabbits, partridges, or mice so we cannot affirm that these animals would be a source of infection for the lynx. On the other hand, all samples in this study were taken from the upper respiratory tract of live animals (except two lymph nodes) while in the previously mentioned studies on NTM in wild boar and red deer a much larger number of samples were taken mainly from lymph nodes or organs with TB-like lesions belonging to hunted animals. This difference in the kind and number of samples would also explain the variety of NTM identified in game animals and the low variety of NTM identified in the lynx. Interestingly, *M. lentiflavum* has also been identified in samples from tracheobronchial washings in humans as mentioned before, so it could be an usual opportunistic mycobacterium of the upper respiratory tract. Finally, the absence of significant differences both in blood parameters and body condition status between healthy and mycobacterium-carrier lynxes as well as the occurrence of lymphadenomegaly in only four lynxes shows that the presence of these two mycobacteria *(M. lentiflavum* and *M. gordonae*) seems not to be detrimental to the health of lynxes.

## 5. Conclusions

Although no MTC has been isolated from the analyzed lynx population, it has been observed that they are a reservoir of environmental NTM, mainly *M. lentiflavum*, the most isolated species. However, the absence of significant differences in both blood parameters and body condition status of healthy and mycobacteria-positive lynxes demonstrates that the presence of these two mycobacteria (*M. lentiflavum* and *M. gordonae*) seems not to be detrimental to lynx health.

## Figures and Tables

**Table 1:** Number of Iberian lynxes sampled in relation to the sex of the animal, age, body condition, and sampling area. Locations are coded by province: CA indicates municipalities in the province of Cáceres, and BA in the province of Badajoz.

Lynx Data	Azuaga (BA)	Don Benito (BA)	Zafra (BA)	Navalmoral de la Mata/Plasencia (CA)	Logrosán (CA)	Mérida	Total
Sex							
Male	14	3	10	1	-	1	29
Female	13	5	6	7	1	1	33
Age							
Yearlings (<1)	7	1	6	2	-	-	16
Subadults (1–3)	5	3	5	2	1	2	18
Adults (>3)	15	4	5	4	-	-	28
Body condition							
≤2	1	-	-	-	-	-	1
2–3	10	-	6	-	-	-	16
≥3	15	7	8	1	1	2	34

**Table 2:** The effect of mycobacteria presence in hemolysis and blood biochemistry on complete blood count of the lynxes examined in our study. Data are presented as means and standard deviation. WBC—white blood cell. Neu—neutrophil. RBC—red blood cells. MCV—mean corpuscular volume. MCH—mean corpuscular hemoglobin. MCHC—mean corpuscular hemoglobin concentration.

Parameters	Mycobacteria		
Hematology	Presence	Absence	*p*-Value
WBC (10^9^/L)	10.1392 ± 0.9923	10.6708 ± 1.2574	0.32
Neutrophils 10^9^/L	7.7458 ± 1.0682	8.3675 ± 1.2127	0.31
Lymphocytes 10^9^/L	1.6000 ± 0.1892	1.7100 ± 0.1788	0.82
Monocytes 10^9^/L	0.2958 ± 0.0355	0.2592 ± 0.0446	0.99
Eosinophils 10^9^/L	0.4717 ± 0.0527	0.2967 ± 0.0458	0.05
Basophils 10^9^/L	0.0267 ± 0.0054	0.0225 ± 0.0051	0.92
RBC (10^12^/L)	8.6825 ± 0.4233	8.7117 ± 0.4257	0.93
Hemoglobin (g/dL)	12.100 ± 0.4919	12.342 ± 0.4375	0.33
Hematocrit%/PCV%	40.025 ± 1.8812	40.300 ± 1.9909	0.84
MCV (fL)	46.192 ± 0.5460	46.40 ± 0.7918	0.12
MCH (pg) Hb/RBC*10	14.0050 ± 0.1598	14.2885 ± 0.2604	0.15
MCHC (g/dL)	30.3158 ± 0.4441	30.8667 ± 0.5076	0.46
Platelets (10^9^/L)	307.750 ± 29.3330	270.50 ± 19.4533	0.97
Biochemistry			
Glucose (mg/dL)	203.9792 ± 19.0453	146.1958 ± 17.6241	0.29
Urea (mg/dL)	112.550 ± 7.1684	116.350 ± 5.9995	0.49
Creatinine (mg/dL)	1.7700 ± 0.1275	1.5517 ± 0.1243	0.2
Cholesterol (mg/dL)	118.667 ± 13.5503	139.167 ± 12.9468	0.63
Triglyceride (mg/dL)	53.250 ± 11.4290	51.417 ± 12.0519	0.24
Total protein (g/dL)	6.992 ± 0.1406	7.092 ± 0.1097	0.68
Albumin (g/dL)	3.5283 ± 0.0902	3.5992 ± 0.0626	0.85
Globulin (g/dL)	3.4675 ± 0.1286	3.4975 ± 0.1283	0.32
Alb/Glob	1.0325 ± 0.0497	1.0500 ± 0.0526	0.63
Total bilirubin (mg/dL)	0.4517 ± 0.0626	0.4225 ± 0.0533	0.53
Direct bilirubin (mg/dL)	0.2525 ± 0.0835	0.2166 ± 0.4827	0.29
Indirect bilirubin (mg/dL)	0.1992 ± 0.0325	0.2058 ± 0.0166	0.89
Calcium (mg/dL)	10.9417 ± 0.4733	10.3425 ± 0.4585	0.78
Phosphorus (mg/dL)	5.2033 ± 0.3861	5.3817 ± 0.4418	
Sodium (mEq/L)	156.133 ± 1.3982	151.392 ± 2.0453	0.85
Potassium (mEq/L)	4.2583 ± 0.0940	4.3925 ± 0.9834	0.841
Na/K	36.8167 ± 0.7154	34.6508 ± 0.8854	0.07
Chloride (mEq/L)	112.958 ± 1.0244	117.575 ± 1.7664	0.09
Plasma electrophoresis			
Albumin (g/dL)	3.8967 ± 0.1816	4.0842 ± 0.0815	0.17
Alfa 1 globulin (g/dL)	0.3467 ± 0.0811	0.2625± 0.0588	0.89
Alfa 2 globulin (g/dL)	0.4375 ± 0.0355	0.3325 ± 0.0374	0.59
Beta 1 globulin (g/dL)	0.6875 ± 0.0500	0.7242 ± 0.0408	0.67
Beta 2 globulin (g/dL)	0.5558 ± 0.0353	0.5317 ± 0.0316	0.69
Gamma globulin (g/dL)	1.1583 ± 0.0901	1.1492 ± 0.0959	0.77
Alb/Glob	1.5175 ± 0.1131	1.5625 ± 0.0728	0.76

## Data Availability

The datasets generated and analyzed during the study are available from the corresponding author on reasonable request.

## Abbreviations

The following abbreviations are used in this manuscript:

NTM	Non-tuberculous mycobacteria
TB	Tuberculosis
MAC	*M. avium* complex
HSP65	65-kDa heat shock protein gene
CDV	Canine distemper virus
SuHV-1	Aujeszky’s disease
FeLV	Feline leukemia virus
RBC	Red blood cell
MCV	Mean corpuscular volume
MCHC	Mean corpuscular hemoglobin concentration
WBC	Differential leukocyte count
ALT/GPT	Alanine aminotransferase
AST/GOT	Aspartate aminotransferase
